# Human PERK rescues unfolded protein response-deficient yeast cells

**DOI:** 10.17912/micropub.biology.000592

**Published:** 2022-06-28

**Authors:** Wei Sheng Yap, Guillaume Thibault

**Affiliations:** 1 School of Biological Sciences, Nanyang Technological University, Singapore, 637551; 2 Mechanobiology Institute, National University of Singapore, Singapore, 117411; 3 Institute of Molecular and Cell Biology, A*STAR, Singapore, 138673

## Abstract

Protein folding and quality control is tightly regulated at the endoplasmic reticulum (ER), and its disruption is associated with many diseases. In eukaryotes, the accumulation of unfolded protein in the ER is sensed by the three sensors, IRE1, PERK, and ATF6 to activate the unfolded protein response (UPR) to restore ER homeostasis. However, uncoupling the sensing of each sensor and their respective downstream pathways has been challenging as the absence of one is compensated by the remaining two sensors. Here, we report a fully functional human PERK (hPERK) chimeric protein expressed in *Saccharomyces cerevisiae* that could be used for high throughput screen to identify new PERK inhibitory or activating compounds as well as to characterize the PERK stress sensing mechanisms.

**Figure 1. Human PERK chimeric protein fully rescues yeast ire1 Δ growth defect upon ER stress:**
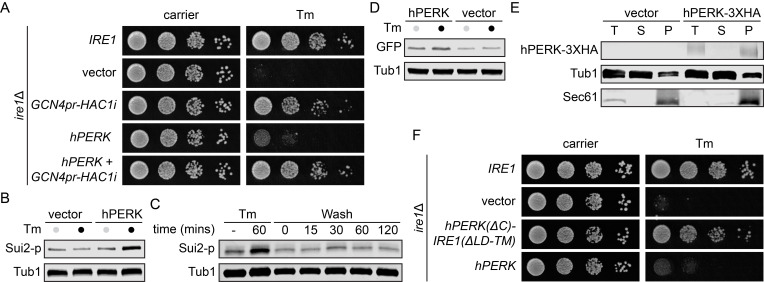
**A** , Strains were grown at 30ºC, and serial dilutions of the culture were spotted onto plates with or without tunicamycin (Tm). **B** , Immunoblot of phosphorylated Sui2 (Sui2-p) from *ire1* Δ mutant cells expressing hPERK incubated 1h with Tm. **C** , Immunoblot of Sui2-p from hPERK-expressing yeast cells were incubated with Tm 1h followed by washout. **D,** Immunoblot of GFP levels driven by yeast promoter *GCN4* in yeast cells expressing hPERK treated with Tm, when indicated. **E** , Microsomes were isolated from the indicated cells. A portion was kept as the total fraction (T), and the remaining was subjected to centrifugation at 30,000 x *g* . Supernatant (S) and membrane pellet (P) fractions were collected and analyzed by immunoblotting. **F** , Strains were grown at 30ºC, and serial dilutions of the culture were spotted onto plates with or without Tm. C, cytosolic domain, LD, luminal domain, TM, transmembrane domain.

## Description

Stress pathways monitor intracellular systems and deploy a range of regulatory mechanisms upon stress. One of the best characterised pathways with wide implications in disease, the unfolded protein response (UPR), is the endoplasmic reticulum (ER) guarding to maintain homeostasis. In eukaryotes, the UPR comprises of three highly conserved transducers leading to the regulation of hundreds of targets by activating UPR-specific transcription factors (Fun and Thibault, 2019). Developed UPR inhibitors to treat diseases have serious potential long term side effects on the functions of the pancreas, the immune system, and the liver as the UPR programme is too broad to be inhibited from the upstream players (Hetz et al., 2013). Additionally, the inhibition or deletion of one of the three ER stress transducers, IRE1, PERK, and ATF6, leads to a compensatory mechanism from the remaining two ER stress transducers. This phenomenon may complicate a search for new ER stress transducer inhibitors. Here, we report a fully functional human PERK (hPERK) chimeric protein expressed in *Saccharomyces cerevisiae* that could be used for high throughput screen to identify new PERK inhibitory or activating compounds.

The sole and highly conserved UPR transducer in yeast, Ire1, is essential to overcome ER stress (Cox et al., 1993). ER stress induces the oligomerization of Ire1 followed by trans-autophosphorylation. Once activated, the Ire1 cytosolic endonuclease domain splices *HAC1* mRNA which is in turn translated into an active transcription factor. Hac1 upregulates hundreds of genes to restore ER homeostasis (Mori et al., 1996, Sidrauski et al., 1996, Travers et al., 2000, Thibault et al., 2011). In higher eukaryotes, PERK is similarly activated by the accumulation of unfolded proteins in the ER lumen as yeast Ire1. In turn, activated PERK phosphorylates eukaryotic translation initiation factor 2 subunit alpha (eIF2α). Phosphorylated eIF2α (eIF2α-P) inhibits protein translation while enhancing the translation of the activating transcription factor 4 (ATF4). Yeast translation initiation suppressor *SUI2* encodes the alpha subunit of eIF2. Similarly in yeast, phosphorylated Sui2 inhibits protein translation while enhancing the general control transcription factor Gcn4. Gcn4 mediates the response to amino acid starvation.

First, we asked if hPERK can replace yeast Ire1 ( *ire1* Δ) during ER stress (Fig. 1A). As expected, heterologous expression of hPERK only partially rescued the growth defect of *ire1* Δ during tunicamycin (Tm)-induced proteotoxic ER stress. Expression of *HAC1* spliced mRNA ( *HAC1i* ) under the control of *GCN4* promoter ( *GCN4pr-HAC1i* ) rescued *ire1* Δ growth defect upon ER stress, suggesting that the basal expression level of *GCN4pr* -regulated *HAC1i* is sufficient to confer ER stress resistance independently of hPERK. Next, to validate if the partial rescue from ER stress in hPERK-expressing yeast cells is caused by protein translation inhibition as in human cells, we measured the levels of phosphorylated Sui2 (Sui2-p) in the absence or the presence of Tm in *ire1* Δ expressing hPERK (Fig. 1B). We observed increased Sui2-p levels upon Tm-induced ER stress, suggesting that hPERK senses the accumulation of unfolded proteins in the ER and phosphorylates its downstream target, Sui2. To assess the ability of hPERK to be deactivated in yeast, we performed a Tm-washout assay and measured Sui2-p protein levels. We detected a drastic dephosphorylation of Sui2 immediately following the Tm washout (Fig. 1C). To monitor the downstream activation of hPERK in yeast, we engineered a GFP reporter driven by the yeast promoter *GCN4* to mimic human ATF4 translation upon ribosome bypass of the upstream inhibitory uORF mediated by the phosphorylation of eIF2α (Fun and Thibault, 2019). An increase in GFP level upon Tm incubation was observed only in hPERK cells (Fig. 1D), confirming the functionality of hPERK to sense proteotoxic stress in yeast. To validate the integration and localization of hPERK into yeast ER membrane, we isolated the microsome and probed for the presence of hPERK. As expected, hPERK was present in the pellet fraction, together with the ER-localized transmembrane protein Sec61, indicating proper integration (Fig. 1E). Finally, to further validate the functionality of hPERK in yeast, we generated a fusion protein containing the luminal and transmembrane domains of hPERK [hPERK(ΔC)] to the cytosolic domain of yeast Ire1 [ScIre1(ΔLM-TM)]. hPERKΔC-Ire1(ΔLM-TM) which was sufficient to rescue the growth defect of *ire1* Δ subjected to Tm, indicating that hPERK senses ER proteotoxic stress in yeast (Fig. 1F).

Defective or overwhelmed UPR is associated with diseases which leads to disrupted ER homeostasis and compromised ER function. However, despite the growing knowledge on the UPR in disease progression, identified drugs targeting the UPR is scarce in clinical trials. To close this research gap, a robust and simple high throughput screen would be ideal. Our findings demonstrated that the unfolded protein sensing module of the UPR pathway in yeast can be replaced by that of human. This tool can be used to screen for potential targets that confer ER stress resistance by uncoupling upstream human proteins from the endogenous downstream UPR programme. As one of the simplest eukaryotes, yeast is well-known for its ease of manipulation in the laboratory while retaining many conserved pathways found in human. Unlike in human cells, the sole yeast UPR branch can be switched off without displaying obvious cell defects in unstressed condition. More importantly, the two other compensatory branches of the UPR are absent in yeast. Therefore, yeast Ire1 can be replaced by ER stress sensors of other species to monitor and manipulate downstream responses, providing a tool to build a complete sense-and-response artificial gene circuit. This toolbox will be essential to dissect the human UPR pathways that are often complex yet intricately interwoven.

In this work, we demonstrated that hPERK expressed in *ire1* Δ marginally rescued yeast cells from proteotoxic stress by inducing Gcn4 expression via Sui2 phosphorylation. This inter-species pathway can be exploited to design a set of heterologous genes driven by the endogenous *GCN4* promoter to complete the gene circuit. However, our data suggest that the *GCN4pr* -driven expression is leaky and induction levels by hPERK is low during ER stress (Fig. 1D). Therefore, an artificial transcriptional system such as the use of artificial transcription factors and synthetic promoters might be better suited. For instance, the artificial ZEV yeast promoter (Kotopka and Smolke, 2020) should be a suitable candidate to express human proteins in response to the synthetic UPR sensor circuit. This inducible system should replicate the stress response feedback loop in which the stress-induced signal strength dictates downstream activation magnitude and attenuation if the stress is resolved. Finally, the plasticity of hPERK to be activated or deactivated in yeast according to the ER stress state (Fig. 1C) reinforce the notion that hPERK is a suitable candidate to be combined with an artificial genetic-driven feedback loop.

Previously in yeast, the overexpression of hPERK WT was reported to phosphorylate Sui2 while several missense variants associated with Wolcott-Rallison Syndrome were deficient in phosphorylating Sui2 (Senee et al., 2004). However, the phosphorylation of Sui2 occurs in quiescent cells suggesting that the overexpression of hPERK leads to self-activation without any ER stress. Nevertheless, they could demonstrate that most of the hPERK missense variants are non-functional and thus contributing to Wolcott-Rallison Syndrome. To prevent hPERK self-activation, we expressed hPERK under the control of yeast *IRE1* promoter. More importantly, the chimeric protein hPERK(ΔC)-Ire1(ΔLD-TM) was sufficient to fully rescue proteotoxic stress-induced growth defect of *ire1* Δ mutant cells (Fig. 1F). This chimeric protein may be utilized for mutational analysis to identify key regions for the sensing of misfolded proteins and lipid bilayer stress as previously reported for yeast Ire1 (Halbleib et al., 2017, Ho et al., 2020, Kimata et al., 2004).

## Methods

**Strains and antibodies** . *S. cerevisiae* strains used in this study are listed in Table 1. Strains were prepared using standard transformation protocols. Anti-HA mouse monoclonal antibodies, HA.11 (MMS-101R-1000, Covance), anti-GFP (11814460001, Roche), anti-p-eIF2α (9721, Cell Signaling Technology), and anti-Tub1 mouse monoclonal antibody (12G10, Developmental Studies Hybridoma Bank) were commercially purchased. Anti-Kar2 rabbit polyclonal was a gift from Davis Ng (Temasek Life Sciences Laboratories, Singapore). Secondary antibodies goat anti-mouse IgG-DyLight 488 (35503, Thermo Fisher), goat anti-rabbit IgG DyLight 550 (84541, Thermo Fisher), goat anti-mouse IgG-IRDye 800 (926-32210, LI-COR Biosciences), and goat anti-rabbit IgG-IRDye 680 (926-68021, LI-COR Biosciences) were commercially purchased.

**Plasmids used in this study** . Plasmids used in this study are listed in Table 2. Plasmids were constructed either by restriction or Gibson cloning. All coding sequences of plasmid constructs used in this study were fully sequenced.

**Spotting growth assay** . Cells were grown overnight in 3 ml of selective media at 30ºC and diluted to 0.2 OD _600_ /ml from which three 10-fold serial dilutions were prepared and spotted on selective plates (0.25 μg/ml tunicamycin when indicated). Plates were incubated at 30ºC until the appearance of colonies.

**Microsome isolation** . In brief, cells were grown to early log phase and the equivalent of 50 OD _600_ of cells were harvested. Cells were resuspended in Tris buffer (50 mM Tris Cl, pH7.4, 50 mM NaCl, and 10% glycerol) containing 1 mM phenylmethylsulfonyl fluoride (PMSF) and protease inhibitor cocktail (04693116001, Roche) followed by four 30 s cycles of bead beating. The lysate was transferred to a new tube and combined with a Tris buffer bead wash. Cell debris was removed by discarding the pellet fraction after centrifuging at 800 x *g* for 5 min, 4°C. The supernatant fraction of the lysate was then spun down at 30,000 x *g* for 30 min, 4ºC. The pellet, containing the microsomal fraction, was solubilized in 3% sodium dodecyl sulfate (SDS), 100 mM TrisCl, pH 7,4, 3 mM dithiothreitol (DTT) and incubated at 95°C for 10 min. Proteins from total cell lysate and supernatant fractions were precipitated with 10% trichloroacetic acid (TCA) and spun down 30 min at 18,400 x *g* , 4°C. Proteins were resuspended in TCA resuspension buffer (1 mM Tris, pH 11, 3% SDS) and incubated 10 min at 95°C. Protein loading buffer was added to each fraction. The protein lysates were separated by SDS-PAGE and transferred to nitrocellulose for immunoblot analysis

**Immunoblot** . Cells were grown to an early log phase overnight at 30°C. Tunicamycin was added to a final concentration of 2.5 µg/ml and incubated at 30°C for 1 h, when indicated. Harvested cells were resuspended in 10% TCA followed by the addition of 0.5 mm zirconium beads. Cells were disrupted by two 30 s cycles of bead beating. The lysate was transferred to a new tube and combined with a 10% TCA bead wash. The precipitate was pelleted by centrifugation and vortexed in TCA resuspension buffer (100 mM Tris pH 11, 3% SDS, 1 mM PMSF). The samples were incubated 10 min at 95°C and spun down 15 min at 18,400 x *g* , 4°C. A portion of the extract was separated by SDS-PAGE using a 10% gel and transferred to nitrocellulose. The blots were probed with primary antibodies followed by secondary goat anti-mouse IgG-IRDye 800 (LI-COR) and goat anti-rabbit IgG-IRDye 680 (LI-COR) antibodies. Membranes were washed in TBS and visualized with the Odyssey CLx imaging system (Li-COR).

Table 1. **Yeast strains used in this study**

**Table:** 

Strain	Genotype	Source
W303a	*leu2-3,112 trp1-1 can1-100 ura3-1 ade2-1 his3-11,15*	(Cox et al., 1993)
YGT0112	*MAT* a, *ire1::* *KANMX* , W303 background	(Ho et al., 2019)

Table 2. **Plasmids used in this study**

**Table:** 

Plasmid	Encoded protein	Promoter	Vector	Source
pGT569	Human PERK (hPERK)	*IRE1*	pRS416	This study
pGT570	GFP	*GCN4*	pRS415	This study
pGT571	Spliced Hac1 (Hac1i)	*GCN4*	pRS415	This study
pGT572	3XHA-tagged hPERK	*IRE1*	pRS416	This study
pGT574	hPERK(ΔC)-ScIre1(ΔLD-TM)	*IRE1*	pRS416	This study
